# Developing, Testing, and Implementing a Falls Prevention and Healthy Aging App (Keep-On-Keep-Up) for Older Adults

**DOI:** 10.1093/geroni/igab046.1990

**Published:** 2021-12-17

**Authors:** Emma Stanmore

**Affiliations:** University of Manchester, Manchester, England, United Kingdom

## Abstract

Falls are a common and costly concern for older adults. Digital technologies can offer new, inexpensive approaches to increase access and engagement with falls prevention programmes. Keep-On-Keep-Up is a personalised, falls prevention App with strength and balance exercises plus health literacy games. This study reports on the user-centred design, usability testing and implementation of the KOKU App. Older adults aged 55 years and older in the UK were invited to take part in the study. Data collection included focus groups; baseline and 6 week questionnaires and assessments; semi-structured interviews and one focus group with falls prevention therapists to explore App usability. Thirty older adults were invited to use KOKU unsupervised, 3 times a week for 6 weeks. Data were analysed using thematic content analysis. Focus groups (n=11) with 66 older users and 11 therapists informed development. Thirty older adults (mean age = 75) were recruited for the in-depth testing. Mean SUS score was 71 indicating high usability. Qualitative themes included: ease of use (app usability; iPad properties; exercise presentation), usefulness (physical/psychological benefits; falls education), attitude towards the App and intention to use (technological barriers; flexibility of use; exercise class versus App). Therapists (n=6) viewed the KOKU platform positively and suggested extensions for further progression. No adverse events were reported during the study. This research demonstrates that KOKU is an acceptable and easy to use falls prevention intervention that facilitates older adults’ ability to access falls prevention training at a time, and in a location, that suits them.

